# Rectal Gastrointestinal Stromal Tumour: A Report of a Rare Case and Literature Review

**DOI:** 10.7759/cureus.67898

**Published:** 2024-08-27

**Authors:** Satyanarayana Kummari, Sairam Subburam, Sree Raksha Chokkalingam, Pushpahaas Jamalapuram, Mahipal Rangi

**Affiliations:** 1 Radiology, All India Institute of Medical Sciences, Nagpur, IND; 2 General Practice, Government Medical College, Chennai, IND; 3 Medicine, Government Medical College, Chennai, IND; 4 General Practice, Kamineni Academy of Medical Sciences, Hyderabad, IND; 5 Radiology, MNR Medical College, Sangareddy, IND

**Keywords:** gastrointestinal tumor (gist), colonoscopy, cect abdomen, rectal gist, gastrointestinal stromal tumor (gist)

## Abstract

Gastrointestinal stromal tumours (GISTs), are an extremely uncommon form of different types of gastrointestinal (GI) malignant neoplasms. While GISTs are the most prevalent type of mesenchymal tumours in the GI tract, they are mainly located in the stomach. Gastrointestinal stromal tumours in the rectum are rarely observed. Some individuals may exhibit symptoms such as constipation, pain in the rectum, bleeding per rectum, or palpable growth, while others may be discovered incidentally. The prevalence of GISTs has been increasing, potentially as a result of developments in imaging techniques. In the present case report, we describe a 47-year-old male patient who initially complained of pain in the lower abdomen, rectum, and occasional constipation. A contrast-enhanced CT (CECT) scan revealed a well-defined hypodense, enhancing lesion with a small calcified area at its periphery in the rectum. The lesion caused a significant luminal narrowing of the rectum. During colonoscopy, a mass located in the submucosal region was identified on the side of the rectal wall, approximately 1 cm away from the anus. After performing the biopsy, the specimen was subjected to histological examination, which revealed a spindle cell tumour with a mild cellular appearance. This finding was in line with the diagnosis of a GIST located in the rectum. The purpose of the current case report is to highlight the significance of CT, colonoscopy, and biopsy in promptly identifying rare GISTs in the colon and rectum, emphasising the uncommon occurrence of GISTs along with their typical locations and imaging features.

## Introduction

Gastrointestinal stromal tumours (GISTs) are rare tumours that develop in the gastrointestinal (GI) tract, primarily in the stomach as well as the small intestines. Nevertheless, in exceptional instances, they might be found in the rectum. Rectal GISTs can be detected either incidentally or when they cause symptoms such as constipation, pain in the rectum, and bleeding per rectum [1]. Rectal GISTs carry a greater likelihood of recurrence and are additionally associated with a poor prognosis when compared to the GISTs located in the stomach [2]. To accurately diagnose these tumours, it is essential to maintain a high level of suspicion. It is recommended that persons with rectal GISTs receive a comprehensive therapeutic strategy involving multiple disciplines and undergo long-term monitoring [3-5].

## Case presentation

**Figure 1:**
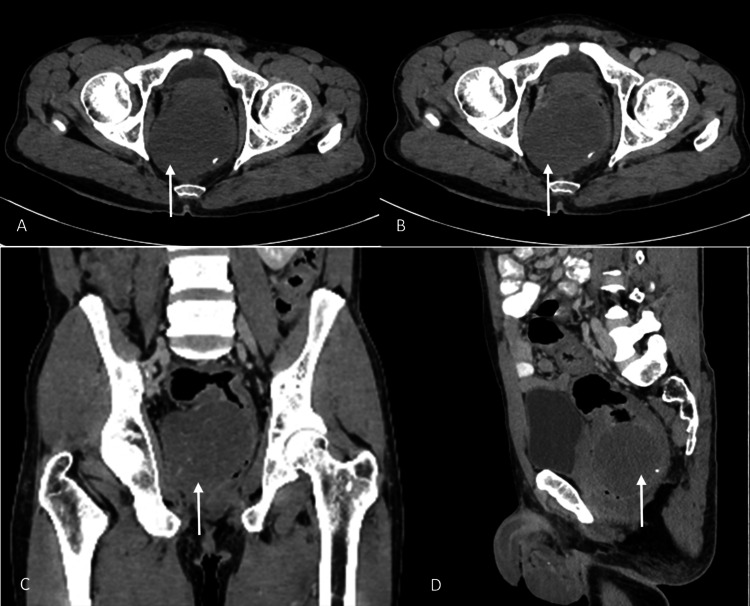
NCCT and CECT scans of the abdomen and pelvis (A) NCCT axial view; (B) axial view; (C) coronal view; and (D) sagittal view of the CECT scans of the abdomen and pelvis show a well-defined hypodense, enhancing lesion with a small peripheral calcification in the rectum (white arrows). The lesion caused a significant luminal narrowing of the rectum. NCCT: non-contrast CT, CECT: contrast-enhanced CT

A 47-year-old male patient without notable comorbidities presented to the medical GI outpatient department for an examination after experiencing occasional constipation and pain in the rectum for a year. Furthermore, he mentioned experiencing rectal pain, which was accompanied by intermittent episodes of frank bleeding per rectum. However, he denied experiencing any additional symptoms. Additionally, he stated that he had never had a colonoscopy before. The patient was recommended to undergo a contrast-enhanced CT (CECT) scan of the abdomen and pelvis. The CECT scan revealed a well-defined, hypodense, enhancing lesion with a small calcified area at its periphery in the rectum. The lesion caused a significant luminal narrowing of the rectum. During colonoscopy, a mass located in the submucosal region was identified on the side of the rectal wall, approximately 1 cm away from the anus (Figure 1).

After performing the biopsy, the specimen was subjected to histological examination, which revealed a spindle cell tumour with a mild cellular appearance. Immunohistochemical examination revealed that the tumour cells exhibited positive expression of CD-34 and CD-117. The diagnosis of a GIST was confirmed. In addition, a Ki-67 assay was conducted, revealing a proliferation rate of less than 1%. To receive additional treatment, the patient was directed to both medical and surgical oncology. To treat the patient, neoadjuvant chemotherapy was administered, and then the tumour was removed through elective surgery. After a year of surgical resection, the patient had a routine sigmoidoscopy, which revealed no signs of recurrence.

## Discussion

Gastrointestinal stromal tumours are uncommon tumours that occur in the alimentary tract and make up approximately 0.1%-3% of all malignant GI tumours [1]. Gastrointestinal stromal tumours are typically identified in older individuals. Both men and women are affected to the same extent. Additionally, recent research indicates that individuals who are extremely obese have significantly greater chances of GISTs compared to people in the general population [6-8].

Gastrointestinal stromal tumours can be seen in any location within the GI tract. After the stomach, which accounts for 60% to 70% of all cases, the small intestine, which accounts for 25 to 30% of all cases, is the most common location. There is also a possibility that they could be found in the colon, rectum, mesenteries, omentum, or retroperitoneum in certain instances. Rectal GISTs make up about 5% of all GISTs and account for about 0.1% of all the tumours of the rectum [2]. The clinical manifestation differs based on the specific location and size of the tumour. These tumours are often discovered inadvertently in numerous individuals. Nevertheless, in certain instances, patients may exhibit symptoms. Rectal GISTs might manifest as pain in the lower abdomen, bleeding per rectum, large bowel obstruction, bowel perforation, or constipation. Occasionally, rectal GISTs may be discovered unintentionally as a palpable growth during a digital rectal examination (DRE) [9].

The evaluation for diagnosing GISTs includes imaging modalities such as ultrasound, CECT, and contrast-enhanced MRI scans, as well as colonoscopy and biopsy. Both CECT and MRI scans are highly effective methods for the diagnosis, staging, and planning of surgeries. They can accurately evaluate the dimensions, configuration, and boundaries of the tumour, as well as detect any potential spread of the disease to distant locations. The histological features of the tumour serve as the basis for the final diagnosis [9,10].

Rectal GISTs are often treated with surgical excision. Lower rectal GISTs that are detected at an early stage can be treated with local excision by transanal, transsacral, or transvaginal approaches. It is possible to treat locally advanced GISTs with neoadjuvant chemotherapy in conjunction with surgical excision [2,11,12]. Gastrointestinal stromal tumours that originate from sites other than the stomach have been associated with less favourable survival rates [13,14]. Specifically, rectal GISTs have been observed to exhibit a significant incidence of recurrence. Hence, it is advisable to conduct extended monitoring of patients with rectal GISTs following their treatment. Typically, it is recommended to do imaging tests like a CT or MRI every three to six months during the initial three to five years after therapy. After that, annual imaging is advised [15]. 

To summarise, rectal GISTs are exceedingly rare and might manifest with diverse signs and symptoms. The current patient reported experiencing intermittent constipation and bleeding per rectum. Colonoscopy, endoscopic ultrasound (EUS), histopathology, and immunohistochemistry were used to confirm the diagnosis of rectal GIST. The patient underwent neoadjuvant chemotherapy with imatinib and a surgical excision.

## Conclusions

Gastrointestinal stromal tumours are uncommon tumours that develop in the GI tract. Typically, these tumours are located in the stomach and intestines. Nevertheless, in exceedingly uncommon instances, they are found in the rectum. Rectal GISTs may be diagnosed inadvertently or may manifest with clinical features such as constipation, rectal bleeding, and lower abdominal pain. Rectal GISTs are more likely to recur and have a poorer prognosis than those located in the stomach. In order to accurately diagnose these tumours, it is essential to maintain a high level of suspicion. In order to diagnose these rare GISTs in the rectum in their early stages, it is recommended to perform a CT, colonoscopy, and biopsy. It is recommended that persons with rectal GISTs receive a comprehensive therapeutic strategy involving multiple disciplines and undergo long-term monitoring.
